# Low high density lipoprotein cholesterol levels and acute coronary syndrome in young patients admitted at a tertiary care facility

**DOI:** 10.22088/cjim.13.4.67

**Published:** 2022

**Authors:** Arpita Chakraborty, CH Sai Kumar, Mukhyaprana Prabhu, Weena Stanley, Ranjan K Shetty

**Affiliations:** 1 Department of General Medicine, Kasturba Medical College Manipal, Manipal Academy of Higher Education, Manipal-576104, Karnataka, India; 2 Department of Cardiology, Manipal Hospitals, Bangalore-560017,Karnataka,India

**Keywords:** Acute coronary syndrome, Coronary artery disease, HDL cholesterol, Myocardial infarction, Gensini score

## Abstract

**Background:**

Coronary artery disease (CAD) is a cardiovascular disease which is related to mortality and morbidity among the Indians predominantly in the older age group. But, recently CAD has been found more often in young population. Hence, our study aims to observe the outcomes based on various categories of high density lipoprotein (HDL) cholesterol levels estimated during admission at the hospital and correlate the levels of HDL cholesterol with severity of CAD as measured by Gensini score.

**Methods:**

A cross-sectional study was conducted in 151 young patients (18-45 years) who were admitted at the hospital with newly diagnosed acute coronary syndrome (ACS). Tests such as electrocardiogram, cardiac enzyme assay, hematologic and biochemical tests including fasting lipid profile levels were taken into account.

**Results:**

There was an inverse relation observed between the number of vessels involvedand HDL cholesterol levels. Those with lower levels of HDL cholesterol were more vulnerable to multi-vessel CAD. However, no association was observed between HDL cholesterol and severity of CAD as measured by Gensini score.

**Conclusion:**

In young patients with acute coronary syndrome and diminished HDL cholesterol levels had a greater number of vessels involved when compared with elevated HDL cholesterol levels group. However, low HDL cholesterol levels had no association with severity of CAD as measured by Gensini score. No statistically significant association was noticed between levels of HDL cholesterol and in hospital mortality /morbidity.

Coronary artery disease (CAD) is recognized worldwide as a significant cause of death (1). It is primarily an illness seen in older population. Most studies indicate that the prevalence rate in young adults of acute myocardial infarction (MI) and CAD with symptoms is around 2-6 percent (4-7). In young adults myocardial infarction leads to greater burden in social life (2-3). Risk factors such as smoking and dyslipidemia predominate among young adults with MI, but they have lesser tendency to develop other confounding factors, such as type 2 diabetes mellitus (T2DM), hypertension (HTN), or past CAD (4-7). Most of the studies involving young patients with acute coronary syndrome (ACS) are reported from the countries in the western region and there are currently no data available on the magnitude, confounding factors, clinical features, and outcomes of such in Asian patients, especially among Indians (4-7). Diminished high density lipoprotein (HDL) cholesterol levels are considered to have association with an elevated risk of coronary events.

The predictive value of diminished HDL cholesterol levels were mainly studied in older (>45 years) patients with CAD of stable nature or those having risk factors for CAD in all previous studies (7). There is very little evidence available on the prognostic value of diminished HDL cholesterol levels in young MI patients. This study therefore, aimed to study the profiles of the risk factors, clinical presentations of young MI patients and to understand the relationship between diminished HDL cholesterol levels and the degree and severity of CAD and performance as measured by Gensini score.

## Methods

This study is a cross-sectional observational study which included 151 young patients (<45 years) admitted under the Cardiology department of a tertiary care hospital in south Karnataka, India with diagnosis of acute MI. Ethics committee approval was taken before the commencement of the study (IEC 613/2014). Informed consent was obtained from all the patients before recruitment. 

The cases who were admitted at the intensive critical care unit (ICCU) under the Cardiology division of the hospital were considered for this study. The inclusion criteria included newly diagnosed cases of young ACS patients within the age limit of 18-45 years. Exclusion criteria included patients who had co-morbidities such as cardiomyopathy, valvular heart disease, previous history of left bundle branch block, acute or chronic liver disease, nephrotic syndrome, hypothyroidism, alcoholics, Cushing’s syndrome, consumption of steroids/ statins and previous history of MI. A comprehensive history of risk factors such as smoking status, T2DM, HTN, obesity and thrombotic disorders were considered. Physical examination, electrocardiogram, cardiac enzyme assay, hematologic and biochemical screening tests and other appropriate investigations as per proforma were performed in all the patients during hospitalization. Diagnosis of several types of ACS and descriptions of data along with the outcome factors were based on the guidelines of clinical data from the American College of Cardiology. In all the patients, laboratory investigations including fasting lipid profile were estimated within a day of admission. In the present study cardiac troponin T (TnT) levels were estimated with immunoassay method in the patients of choice within a day of occurrence of MI. Cardiac troponin T levels > 0.02mg/dl were considered to be elevated. All patients underwent coronary angiography during hospitalization. Severe CAD was considered as stenosis of more than 50% in left main coronary artery or stenosis of >70% in any of the coronary arteries (8).

Acute MI has been identified as elevation of the markers of myocardial necrosis with changes in the electrocardiogram (ECG) which is suggestive of ischemia involving ST elevation or depression, pathological Q waves in ECG and echocardiographic evidence of a recent abnormality in regional wall motion. “Body mass index (BMI) >24.9 kg/ m^2^ was considered as overweight whereas, BMI of 30 kg/ m^2^ was considered as obese”. In the assessment of CAD severity, the Gensini scoring tool was used. The Gensini score was determined from the coronary angiogram, a severity score dependent on the degree of luminal narrowing of each coronary stenosis and its regional significance. Decrease in the diameter of lumen and the radiographic presence of lesions which are concentric, and plaques of eccentric nature were measured. 

“IBM SPSS Version 16 was used for the statistical analysis. Chi Square test was used to compare the categorial variables. It was expressed in terms of frequency and percentages. For a p-value <0.05 statistical significance was considered”. 

## Results

**Table 1:** Baseline characteristics of cardiac risk factors in young patients with MI

**Cardiac risk factors**	**Frequency (%)** **Median (Q1,Q3)**
Smoking	127 (80.1%)
T2DM	57 (37.7%)
Hypertension	47 (31.1%)
Obesity	25 (16.6%)
Thrombotic disorder	3 (2.1%)
Family history of CAD (Yes) (No)	37 (24.4%) 114 (75.6%)
Dyslipidemia	134 (88.7%)
Total cholesterol (mg/dl)	159.5 (128.75,193.25)
Triglycerides (mg/dl)	128 (96,166.75)
LDL cholesterol (mg/dl)	91.5 (65.75,123)

The baseline characteristics such as the cardiac risk factors and the lipid profile values of the patients with MI are described. In our study, in young patients, most common risk factors were smoking (80.1%) and dyslipidemia (88.7%) followed by hypertension (31.1%), obesity (16.6%), diabetes (37.7%) and thrombotic disorder (2.1%). Family history of CAD was found in 37 (24.4%) young patients with MI (table 1). HDL cholesterol levels had a negative association with severity of CAD, i.e. with a decrease in HDL cholesterol levels more number of vessels were involved. A p<0.0001 indicates that low HDL cholesterol predisposes to multiple vessel involvement in young individuals with CAD (table 2). HDL cholesterol did not have relationship with severity of CAD which stated that decline in HDL cholesterol levels did not lead to increase in severity of MI as measured by Gensini score (table 3). 

**Table 2:** Distribution of HDL cholesterol and severity of MI

**Severity of CAD**	**Very low HDL cholesterol (10-29 mg/dl)**	**Low HDL cholesterol (30-39 mg/dl)**	**Normal/High HDL cholesterol (>40 mg/dl)**	**Total**	**P-value**
Single vessel disease (SVD)	24 (22.6%)	60 (56.6%)	22 (20.6%)	106 (70.2%)	<.0001*
Double vessel disease (DVD)	4 (12.1%)	27 (81.8%)	2 (6.06%)	33 (21.9%)	
Triple vessel disease (TVD)	9 (75%)	2 (16.6%)	(8.3%)	10 (6.6%)	

*Chi Square test

**Table 3:** Distribution of HDL cholesterol with severity of MI as measured by Gensini score

**Gensini score**	**Very low HDL cholesterol (10-29 mg/dl)**	**Low HDL cholesterol (30-39 mg/dl)**	**Normal/High HDL cholesterol (>40 mg/dl)**	**Total**	**P-value**
< 50	24 (22.6%)	67 (62%)	17 (15.7%)	108 (71.5%)	0.05
51-100	6 (18%)	22 (66.7%)	5 (15.06%)	33 (21.9%)	
>100	6 (60%)	2 (20%)	2 (20%)	10 (6.6%)	

*Chi Square test

**Table 4:** Distribution of HDL cholesterol and its association with complications in ACS

**Complications**	**HDL cholesterol (normal/high levels)**	**Low HDL cholesterol levels**	**Total**	**P-value**
Patients with complications	22 (14.5%)	109 (72.8%)	131 (72.8%)	0.06
Patients without complications	2 (1.3%)	20 (11.2%)	11.2%)	

*Chi Square test

Number of patients suffering from ACS with complications were more than that without complications. However, significant association was not observed between levels of HDL cholesterol and complications of ACS (table 4). Our study included less number of study population without any complications and could not be categorized according to HDL cholesterol levels as it had very less number of patients under each category. For this purpose, HDL cholesterol categories decreased to 2, one with diminished HDL cholesterol level and the other with normal /high level of HDL cholesterol and the relation between low cholesterol HDL levels and in-hospital complications were observed. No statistical significance was found. However, most of them who had low HDL cholesterol levels had post ACS complications. The mean age of the young patients with MI was 40.5 years. 81 (53.6%) were from 41 to 45 years, 52 (34.4%) at 36-40 years, 16 (10.6%) were 31-35 years and 2 (1.3%) at 26-30 years. Out of the 151 patients, 140(92.7%) were males and 11 (7.3%) were females. Male to female ratio was 12.7. 47.3% of males were in age group 41-45 years, followed by (33.1%) in 36-40 years and 10.1% in 31-35 years. Maximum number of females (6.8%) were from age group 41-45 years, followed by 1.3% in the age group 36-40 years, 0.7% were from 30-35 years and 25-30 years age group each. Out of 151 patients, 79 (52.3%) patients had anterior wall MI, 32 (21.2%) with inferior wall MI, 22 (14.6%) with NSTEMI, and 18 (11.9%) had unstable angina. The most common vessel involved was LAD (5.5 %), 2.4% had insignificant stenosis, 3 % with coronary thrombus and 1.3 % had coronary dissection. Out of the 151 patients, 114 (75.5%) patients had left anterior descending artery involvement, 33 (21.9%) had left circumflex, 57 (37.7%) with right coronary artery, 2 (1.3%) had left main coronary artery involvement. The most common clinical presentations were chest pain (96.7%), followed by dyspnea (12.6%), palpitations (9.9%) and sweating (14%). The most common confounding factors in the patients with younger age were smoking (80.1%), dyslipidemia (88.7%) followed by hypertension (31.1%), obesity (16.6%), diabetes (37.7%) and thrombotic disorder (2.1%). Out of 151 patients with MI, 37 (24.4%) had very low HDL cholesterol levels (10–29 mg/dL), 90 (59.6%) had diminished HDL cholesterol levels (30–39 mg/dL) and 24 (16%) had normal/high HDL cholesterol levels (>40 mg/dL). Arrhythmias (9.6%), congestive heart failure (1.98%), and cardiogenic shock (0.6%) were the problems that were observed to be the most prevalent in patients who were likely to be younger. Maximum patients (88%) were without complications. In the hospital, mortality rate was nil throughout the study period.

## Discussion

MI is a disease which is majorly found in the older age groups and is rare in young people, even though it occurs among Indians at an early age when compared to the population in the Western countries (9). In our study, the mean age of people presented were 40.6 years. Most of the other studies showed almost the same mean age. Wong et al. stated that three subgroups comprising of Malaysians, Chinese and Indians respectively, in Indian group the mean age noticed was 39.9 years in comparison to Malaysians (1.25 times risk) and Chinese (0.7 times risk). Indians were 3 times more prone to develop acute MI before 46 years of age (9). A study stated that 65 patients of young MI were from Indian sub- continent and the mean age found was of 40 years stating the increased prevalence of acute MI in subjects of younger age group. Early age of onset might be due to the increased prevalence of the confounding factors such as smoking status, dyslipidemia and obesity among the young people. In this study majority of the patients (53.6%) fell under 41-45 years age group (10). In a study by Sricharan et al., majority (70%) of the patients were from 35 to 40 years age group (10). In our study, very few (1.3%) patients were below 30 years. It was observed that even in the young adults that constituted our study population, there was a trend of increase in average age at which MI occurred. The same observation was also noticed in past studies.

One of the recognized major coronary risk variables in patients with MI of younger generation is smoking. The most notable confounding factor after dyslipidemia (87.4%) was smoking or any form of tobacco consumption (80.1%) followed by T2DM (37.7%), HTN (31.1%), obesity (16.6%) and thrombotic disorders (2.1%). In a study, most common risk factor was smoking (76.9%), dyslipidemia (33.8%), and hypertension (18.5%). Sricharan et al., stated that the most notable confounding factor was smoking (77.2%), dyslipidemia (54.5%), and HTN (45%) in his study (10). A study stated that the most common risk factor was smoking (70%) (10). In the previous studies, it was revealed that among the acute MI patients under the age of 40, 73 to 90 percent admitted having smoked in the past. Several other studies also showed high tobacco use/smoking rates up to 80 percent among young adults (12-14). Smoking increases the concentration of fibrinogen and aggregation of platelets, results in decreased activity of fibrinogen, impaired flow in coronary arteries and elevates vasospasm (15-16). 

Many of the patients suffering from acute MI had diminished HDL cholesterol levels during admission. One of the objectives of our study was to see the relation of HDL cholesterol with severity. In our study which includes 151 patients, there were more number of patients suffering from single vessel disease and very few patients suffering from triple vessel disease. Between the number of arteries involved and HDL cholesterol levels, a significant relationship was identified. With decrease in HDL cholesterol levels, the percentage of people with severe CAD increased (p<0.001). Those with lower levels of HDL cholesterol were more vulnerable to multi-vessel CAD. Similar observation was seen in study by Vaidya et al. (16). Those with lower HDL cholesterol levels had a higher tendency for multi-vessel CAD among all MI patients with younger age who had undergone diagnostic coronary angiography. Similar finding was obtained in a study by Chua et al (17). Few studies showed negative correlation with HDL cholesterol levels. But our study did not find any association with HDL cholesterol and Gensini score. For patients with ACS, diminished HDL cholesterol levels tend to be a major risk factor. The potential mechanisms for correlating highly diminished HDL cholesterol levels with unfavorable prediction of ACS for a short period of time are unclear. Very highly diminished HDL cholesterol post ACS can act as a marker for more severe CAD and inflammation during the index hospitalization and might lead into rupturing of plaques and death.

Out of 151 patients, 20 people had in-hospital complications. No deaths were observed during the time period of the study. The relationship between HDL cholesterol and hospital death rates and morbidity was statistically not significant. In a study by Acharjee et al., there was a strong relation observed between HDL cholesterol and in hospital morbidity and mortality (18). Similar finding was obtained in one of the studies by Chua et al. (17). In the above studies, the study population was large and when these people with in-hospital mortality/morbidity were categorized based on HDL cholesterol levels, there were significant number of variables under each group. But, our study included a very low number of study population with very less complications which could have affected the results. In our study HDL cholesterol had no significant association with severity of MI as measured by Gensini score which was similar to the findings of Shou et al (19). This study stated that in patients suffering from coronary lesions significant correlation was not found between HDL cholesterol levels and Gensini scores and HDL cholesterol was also not found to be a marker for predicting coronary lesions with modified Gensini scores (19). In a study by Xu et al. stated that large HDL cholesterol was negatively correlated with Gensini score (r= -0.191, p=.005*) and small HDL cholesterol was positively correlated with Gensini score (r= 0.145, p=.023*) in CAD patients which was contrary to the findings of our study (20).

This study did not include a group of older adults. So, the comparison of severity of CAD between the younger and older adults could not be done. Since, it was a time bound study, sample size was also too small. Since, sample size was limited, some of the variables were small in the subgroups which could have affected the power of the study.

In conclusion, in young adults with acute coronary syndrome and diminished HDL cholesterol levels had a greater number of vessels involved when compared with elevated HDL cholesterol levels group. However, low HDL cholesterol levels had no association with severity of CAD as measured by Gensini score. A significant association was not noticed between levels of HDL cholesterol and in hospital mortality /morbidity. 
